# Social networks and health behaviors during the COVID-19 pandemic: a qualitative study among older adults in the Netherlands

**DOI:** 10.1007/s10389-023-01947-9

**Published:** 2023-06-08

**Authors:** Lisanne C. J. Steijvers, Floor Leeferink, Stephanie Brinkhues, Christian J. P. A. Hoebe, Nicole H. T. M. Dukers-Muijrers

**Affiliations:** 1 Department of Social Medicine, Care and Public Health Research Institute (CAPHRI), Maastricht University, Maastricht, the Netherlands; 2 Department of Sexual Health, Infectious Diseases and Environmental Health, Living Lab Public Health, South Limburg Public Health Service, Heerlen, the Netherlands; 3 Department of Knowledge and Innovation, South Limburg Public Health Service, Heerlen, the Netherlands; 4 Department of Medical Microbiology, Infectious Diseases and Infection Prevention, Care and Public Health Research Institute (CAPHRI), Maastricht University Medical Centre (MUMC+), Maastricht, the Netherlands; 5 Department of Health Promotion, Care and Public Health Research Institute (CAPHRI), Maastricht University, Maastricht, the Netherlands

**Keywords:** Social relationships, Social network structure, Social network function, Social support, Health behavior, COVID-19 pandemic

## Abstract

**Aim:**

Social networks, all social relationships that people have, may influence people’s health behavior and well-being, which was evaluated in this qualitative study in older adults. Furthermore, we evaluated people’s needs for strengthening social networks.

**Subject and methods:**

For this qualitative study, semi-structured interviews were conducted between May and July 2021 among 24 adults aged 60 years and older.

**Results:**

Respondents provided information on social network structure (number and types of relations) and function (social support). They received informational support from friends, emotional support from their partner/spouse, and all types of support (including practical support) from family. Respondents stated that their health behavior was mainly influenced by a partner/spouse. Family and friends were mostly for socializing. To strengthen networks, in-person bilateral or small group interactions were preferred.

**Conclusion:**

Family and friends were important social supporters and positively influenced health behaviors. This study emphasizes the importance of social networks in health promotion.

## Background

Social interactions are key to people’s well-being, resilience, and physical, mental, and social health (Hakulinen et al. 2016; Valente 2015). Globally, social interactions strongly decreased during the COVID-19 pandemic. People contacted fewer social network members and had less in-person contact (Freedman et al. 2022; Liu et al. 2021). Moreover, in the Netherlands social networks have become smaller, with fewer emotional and practical social supporters (Steijvers et al. 2022; Völker 2023). A lack of supportive social interactions is associated with the onset and progression of diseases (Brinkhues et al. 2017). The loss of social interactions may result in loneliness and other negative health conditions, with associated unforeseen health consequences in the longer term (Leigh-Hunt et al. 2017). This is especially relevant in older individuals (Armitage and Nellums 2020), who comprise a rising share of our societies. Loneliness among older people is seen as a growing public health problem and globally 20–34% of older adults are lonely (World Health Organisation 2021). Older adults usually have fewer relationships than younger persons (Kemperman et al. 2019). During COVID-19 lockdowns in the Netherlands, more than 60% of the older adults experienced less contact with their family and friends (Baâdoudi et al. 2021).

The web of social interactions and personal relationships, a social network (Oxford University Press n.d.), can be described by its structure and function (Antonucci and Akiyama 1987; Berkman and Glass 2000). Structural social network aspects are, for example, the number of relationships (social network size), type of relationships, mode of contact, the proximity of the social network members, and the frequency of social contacts. Functional social network aspects include social support such as informational social support (advice), emotional support (when a person experiences discomfort or wants to discuss important matters), or practical social support (jobs around the house and or help when a person is ill) (Berkman and Glass 2000; Brinkhues et al. 2017). Relationship types (social network structure) are related to different types of received social support (social network function) (Holt-Lunstad 2023). In older adults, a relationship with their children is, for instance, strongly related to the provision of practical support and ties with friends and relatives to the provision of emotional support (Stuifbergen et al. 2008)). Also, a higher frequency of contact is associated with more provision of social support in older adults (Bui 2020). However, there is a lack of knowledge on types of social interaction and the structure and function of older adults’ social networks during the COVID-19 pandemic.

**Fig. 1:**
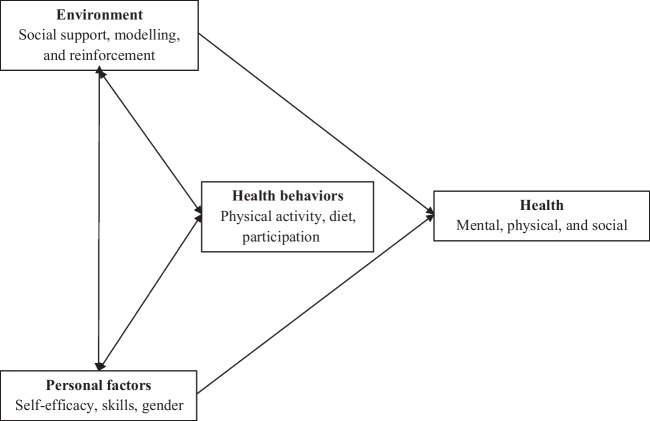
Social cognitive theory and its interaction with health

Social networks influence health and health behaviors such as physical activity (Hailey et al. 2022; Flatt et al. 2012), and dietary behavior (Harmon et al. 2016; Vesnaver and Keller 2011). The mechanisms by which social networks operate on health behaviors could be illustrated by the Social Cognitive Theory (SCT) (Fig. 1) (McAlister et al. 2008; Schunk and DiBenedetto 2020). This theory explains behavior through an interaction between personal, environmental, and behavioral factors (McAlister et al. 2008). A person’s social network is part of their social environment (Fig. 1). The environment can influence a person’s behavior, for instance via receiving social support (Khami et al. 2020), by being a model regarding health behaviors, or through facilitation, i.e., providing access to certain resources (McAlister et al. 2008). In addition, reinforcements such as incentive motivation coming from the environment of the person, can influence their health behaviors (Lee and Lim 2015).

The interplay between environment and health behavior is also depicted in the concept of positive health (Huber 2019). Within the concept of positive health, several dimensions are defined, including mental well-being, meaningfulness, quality of life, and social participation. The latter consists of social contacts, perceived social support, conviviality, and belongingness (Huber et al. 2011, 2016). Social participation is key to resilience in health and is seen as a key component in successful aging (Ghazi et al. 2017).

Several initiatives have been taken by the Dutch government to include social networks when improving older adults’ well-being (RIVM 2021). To enable tailoring, research from the perspective of the older adults themselves is preferred. Therefore, in this qualitative study, we examined how they value their social network structure and function, and what role the network played in their health behavior during the COVID-19 pandemic. In addition, we aimed to assess needs and preferences regarding strengthening their network, such as by adding new social relations or enhancing social support from their existing social relations.

## Data and methods

### Ethics statement

This study was approved by the Medical Ethical Committee of the University of Maastricht (METC 2021-2710). Participants were recruited from people 60 years and older independently living in the province of Limburg, Netherlands, who participated in the Corona Study Limburg (COL) (Pagen et al. 2022). Invitees gave permission to be approached for research purposes.

### Study design

In this descriptive qualitative study, semi-structured interviews were conducted via phone or video calls.

### Study sample

Participants were aged 60 years and older. For the sampling, a quota sampling method was used based on the sex and age of older adults; 100 women and 100 men were invited to participate in the study. The participants were contacted by email and telephone. Of invitees, 25 (12,5%) decided to participate; one participant withdrew, resulting in a final sample of 24 participants.

### Data collection instruments and measures

Data collection took place in May–July 2021. A female researcher held the interviews, and a second female researcher was present to observe and co-guide the interview. Semi-structured interviews were conducted via phone or video calls, audio-recorded for transcription purposes, and lasted approximately 45–60 minutes each. In addition, notes were made during the interview by the researchers. An interview guide containing several topics (Appendix A) and corresponding questions was developed a priori and updated after each interview. This guide was developed based on the theoretical model, literature, and findings from the Social Network Assessment in Adults and Elderly (SaNAE) study ( Steijvers et al. 2021; Steijvers et al. 2022). The interview guide provided a certain direction for the conversation; however, there were also opportunities for new information (Stuckey 2013). The guide was pilot tested on older people and co-researchers.

A topic list was created, which can be found in Appendix A. Topics were based on the research objectives: (I) structural social network aspects, (II) functional social network aspects, (III) impact/influence of the social network, and (IV) intervention. These items were the basis for the coding tree. Structural social network aspects such as type of relationships, mode of contact, and proximity of the participants’ social network members were questioned. Functional social network aspects included different types of support. The impact/influence of the social network was questioned by asking about exercising, diet, and social participation. Also, feelings of loneliness were assessed. Finally, we investigated if and how participants would like to see an intervention that can positively strengthen their social network, by inquiring about important aspects of a social network, the participant’s satisfaction with their social network, and their preferences regarding the mode of contact, and the intervention itself. In addition to the main topics, the participants’ demographics were asked at the beginning of the interview, including age, marital status, living situation, and employment status, but also other personal information such as health status.

Before the interviews, the interviewee was sent a written informed consent form, including all the study information. At the beginning of the interview, the aim and purpose of the study were explained once more, and the participant needed to sign the informed consent form and give verbal consent as well. Participants received a €10 reimbursement voucher for participating. In total, 24 interviews were conducted, reaching data saturation after 21 interviews (Braun and Clarke 2021).

### Data analysis

Audio recordings were transcribed verbatim by an external transcription service company. The audio transcripts were transported to ATLAS.ti 9. A thematic analysis was performed using an interplay between an inductive (bottom-up) and a deductive (top-down) approach (Braun and Clarke 2006). To conduct the thematic analysis, the six-phase method by Braun & Clarke was followed. These six phases are 1. Familiarizing yourself with the data, 2. Generating initial codes, 3. Searching for themes, 4. Reviewing themes, 5. Defining and naming themes, 6. Producing the report (Braun and Clarke 2006). This way of coding brings the advantage to explore without prejudice but keeping the theories in mind (SCT). Because in-depth research on the preventive potential of social networks and health behaviors of older adults, during the COVID-19 pandemic, is limited, this coding process was considered most fitting. To ensure objectivity, three research team members coded the transcripts independently. Discrepancies between codes were discussed within the research team until a consensus was reached.

## Results

### Demographic characteristics

The study sample consisted of diverse older people according to sex, age, marital status, employment situation, and living situation (Table 1).

**Table 1:** Demographic characteristics of participants (*n* = 24)

Demographic characteristics	n (%)
Sex	
Female	12 (50)
Male	12 (50)
Age (years)	
60–64	9 (38)
65–69	10 (42)
70–74	3 (13)
75–79	1 (4)
80+	1 (4)
Marital status	
Married	13 (54)
Widowed	5 (21)
Relationship	4 (17)
No relationship	2 (8)
Employment situation	
Working	7 (29)
Retired/not working	17 (71)
Living situation	
Living alone	7 (29)
Not living alone	17 (71)

### Social network structure

Social network size and diversity varied between participants. Most participants mentioned several different social network members throughout the interview, whereas other participants repeatedly mentioned the same persons. Most participants mentioned multiple types of relationships, such as partner/spouse, family, neighbors, colleagues, acquaintances, and formal care providers, such as the GP. They also mentioned group memberships, such as clubs or organizations: *“We have very good family ties, I have a lot of friends, and acquaintances. I am a volunteer at [voluntary organization 1], so I also visit a lot of people and make many phone calls.” (P7, female, 75–79y, retired/not working, living alone)*. However, some participants mentioned only a few different relationship types, for example, their partner/spouse or family.

Regarding mode of contact, several participants mentioned having contacted certain network members exclusively via phone or online contact: *“I send a lot of emails to friends to ask how they are doing, but I also call.” (P19, male, >80y, retired/not working, not living alone)*. For some participants, online contact was a result of the COVID-19 measures of social distancing, contact reduction, and lockdowns. Exclusively contacting network members by telephone or online chatting was not perceived as fulfilling the need for contact for every participant. They mentioned still meeting with their social network members in person: *“At first we strictly kept our distances from each other, but after three months we still got together with one or two persons, the children, because it was otherwise impossible to sustain.” (P17, female, 70–74y, retired/not working, living alone)*.

The geographical proximity of social network members varied between participants. Some participants had most of their social network members living nearby, while others had to travel by car to see most of their friends and family. The proximity of the network members determined the mode and frequency of contact. Network member(s) living further away were contacted more often via phone or chat, whereas network members living nearby were more often contacted in person: *“One friend lives a bit further away and one friend lives a village further away, so then it is easier to go there, in-person.” (P1, female, 60–64y, working, not living alone)*. Proximity also determined received social support. Participants stated to have more frequent contact with social network members who live nearby, and that practical support received was provided by a family member living nearby.

### Social network function

#### Informational support

Most participants received informational support (advice or counseling) specifically from their friends and family*: “I have a lot of friends here in the village, so if I needed advice I would usually go to my friends here nearby or my brother, depending on the topic.” (P6, male, 65–69y, retired/not working, living alone)*. Participants also sometimes received informational support from their partner/spouse, acquaintances, and a health care professional. Few respondents mentioned that they received informational support from neighbors or colleagues.

#### Emotional support

Emotional support regarding important decisions and discomfort was mainly received from family and partner/spouse: *“Very personal stuff I discuss with my wife or my brother.” (P8, male, 65–69y, retired/not working, not living alone)*. Friends were contacted sometimes when important decisions needed to be made or important matters needed to be discussed. Sometimes, colleagues, neighbors, acquaintances, and healthcare professionals were also contacted. Health care professionals were mainly contacted for medical decisions: *“My wife’s caregiver comes over regularly and you can also discuss a lot of things with that young lady, fortunately.” (P15, male, 60–64y, retired/not working, not living alone)*.

Several participants mentioned that the emotional support received from acquaintances was unexpected yet very pleasant. *“And even for the social contacts that are not very close, such as that acquaintance. Those are people who regularly text and say how are you? And are you okay? Those are things that do good. Or people who suddenly send a card unexpectedly. And those are not those social relationships that you see very often but that do think of you. And those are very important things in life. I have experienced that the last two years.” (P3, female, 65–69y, retired/not working, living alone)*. In addition, most of the participants stated it was difficult to discuss important matters by telephone or online messaging. However, some could discuss these matters by telephone as well. Most participants use online messaging, but mainly for quick, short messages. For more serious matters, telephone and especially in-person contact are preferred. Few participants had not felt the need to discuss important matters with other people.

#### Practical support

Practical support, regarding doing jobs around the house and regarding help when ill, was mostly received from family. Both types of practical support were also received from friends, a partner/spouse, acquaintances, and neighbors. However, most participants did not need help when doing jobs around the house and sometimes confidence in taking care of themselves was addressed*: “I also try more and more things myself which I used to think no, I cannot do that, like putting together cabinets when you bought something new or something like that. In the past, we did that together, and then I thought no, I cannot do that alone. But you can do quite a lot yourself if you try.” (P3, female, 65–69y, retired/not working, living alone)*. Practical support when being ill was sometimes received from (former) colleagues and a health care professional. Some participants also mentioned that they did not need practical help when they were ill as they were still self-reliant.

#### Multidimensional support

Multidimensional support included emotional support with either informational or practical support, and sometimes all three types of support. The social network members from whom the respondents received multidimensional support were usually family or friends and in some cases acquaintances or a partner/spouse: *“If I needed advice on something I would usually contact my children or close family members such as my sisters or brother. … And if I need to talk about important topics or make decisions, I would also talk to my sisters.” (P22, female, 65–69y, retired/not working, not living alone)*. From family other than the partner/spouse the respondents usually received all three types of social support (only one person received multidimensional support from exclusively the partner/spouse), or only emotional and practical support. From the partner/spouse or friends, the respondents typically received emotional support in combination with either practical or informational support.

### Influence of social networks on lifestyle

#### Exercise

Many participants exercised together with social network members, as this motivated them to exercise more often, and it was more pleasant to exercise together: *“I am at such a sports club, and I have been going there with my sister and daughter. They both chose something different, so I am going there alone. But yes, I know plenty of people. So, I do like sports in a group more than on my own. I also think it stimulates.” (P11, female, 60–64y, working, not living alone)*.

Neighbors providing informational support *(“exercising is a good preparation for upcoming surgery”)* resulted in exercising more often for one participant. During the COVID-19 lockdown, sports facilities were closed, and many participants reported going for a walk instead. Most went walking together with social network members and started to find this more fun than walking alone. Some participants mentioned to have exercised less frequently because they could not exercise in a group or a physical environment due to COVID-19 restrictions: *“We also had a sports club with some friends, but that has not been for two years. So, then I exercise a little less.” (P10, male, 65–69y, retired/not working, not living alone)*.

Participants exercised together, mostly with their partner, but also with family, friends, or acquaintances. A few mentioned to prefer exercising alone because others slowed them down, or it was less safe (cycling with multiple people). Also, one respondent mentioned that a close social network member did not like to exercise, which made it more difficult for the respondent to exercise.

#### Diet

Many participants stated they decided for themselves what to eat and that they usually tried to eat healthy. They also mentioned that social network members (partner/spouse, family, or health care professional) made it easier to eat healthy by providing emotional support to eat healthy, informational support on nutrition, practical support by cooking a healthy meal or buying healthy groceries. Women mainly provided such support and influenced their male partners. In some cases, there was a specific reason mentioned for the need to eat healthier, such as a health condition or a higher risk of developing diabetes or cardiovascular diseases. Some also reported that family and friends had made it more difficult to always eat healthy:* “I know what I can and cannot eat because of my [disease X], so I try to keep that in mind and not eat greasy food every day, but when I’m at a café and someone says let’s order some fried food, I’ll be the last to say no.” (P24, female, 65–69y, retired/not working, living alone)*.

### Influence of social networks on aspects of health

#### Social participation

Social network members, usually family and friends, and sometimes with acquaintances, a partner/spouse, neighbors, or (former) colleagues, were important for conviviality. Some mentioned finding it hard to do fun things alone: *“We always went on holiday together quite regularly. I think that is an issue now because I would not be quick to say I am going on holiday alone. I find that difficult, and then I am very much thinking about how I should approach that later, well, to go somewhere again. Because to go all alone, that step is a bit too big for me.” (P3, female, 65–69y, retired/not working, living alone)*. Some did not want to do fun things anymore when social interactions were restricted due to COVID-19.

Others were a member of an association such as a carnival association, a music association, or a sports club. However, active participation in these physical location-based associations was not possible due to COVID-19. Some participants also mentioned that the bond of trust they had with their colleagues was a reason for them to maintain working, instead of retiring.

#### Loneliness

Some persons (both persons who live alone and those who do not live alone) experienced feelings of loneliness because of too few social relationships or contacts, or a lack of a supportive social network. Some were lonely and desired more social contact. Loneliness was particularly related to the COVID-19 pandemic and its lockdowns because fun activities and contacts were not possible:* “Everyone was scared, nobody comes over and you have nowhere to go. Then you feel, abandoned I don’t want to say, but isolated. Still isolated and sometimes in the morning I thought, why should I get up, I might as well turn around one more time. Not that I did. But I have never had that thought before, but I did have that in the spring when the weather was bad for a long time, and we could not do anything. That was difficult, especially when you had a day when you had not heard your voice yet. So, I was happy when some things were allowed again.” (P14, female, 65–69y, retired/not working, living alone)*. Others enjoyed the time they got to spend alone and had more time to read: *“I did not experience any feelings of loneliness. On the contrary, I quite enjoyed the time I got to spend all by myself during the lockdowns.” (P6, male, 65–69y, retired/not working, living alone)*.

At moments when participants were lonely or desired social contact, they sometimes felt uncomfortable seeking social contact within their social network because they did not want to bother them. Also, some participants feared becoming lonely in the future because they have seen this happening with people in their social network.

### Preferences for strengthening social networks

#### Important aspects of social networks

Participants reported that an important value of the social network was to be offered a sympathetic ear (emotional support), receive help when needed (practical and informational support), conviviality, and trust. Another important function of the social network was to have reciprocal contact, which is an effort coming from both sides of the relationship to keep contact and to reciprocally be able to talk about personal matters: *“People surrounding me should offer a sympathetic ear, and have to take you seriously when you have something to say or are struggling with something, but also that there is mutual contact so that people ask about you as well and ask you to go somewhere together.” (P13, male, 65–69y, retired/not working, living alone).* Other important aspects mentioned were respect, good communication, physical contact, a safe feeling, attention, being taken seriously, and honesty.

#### Satisfaction with social network

Most participants mentioned that important social network aspects were present within their network, and they reported overall being satisfied with their network. Some participants mentioned having a larger social network with many contacts. Others mentioned being satisfied with the support that they received from their small network: *“I do not have a very large social network, but the quality is good.” (P5, male, 60–64y, retired/not working, not living alone)*.

Superficial relationships or work-related relationships were reported mainly for socializing and built on conviviality. Participants reported enjoying these relationships, though they would not share personal matters with such relation types, these relations do contribute to their feelings of satisfaction with the network: *“Yes, they are not such friends with whom you discuss everything. That is more of the fun side of life. But I am not discussing problems or other things with them.” (P3, female, 65–69y, retired/not working, living alone)*.

Participants however mentioned being mainly satisfied with the social network they had before the COVID-19 period*: “No, I like it the way we had it before corona, I think it is fine that way. With my friends and acquaintances and family, I think that is fine.” (P22, female, 65–69y, retired/not working, not living alone)*.

Although sometimes in-person contact was replaced by some other remote type of contact, participants missed the previous in-person (face-to-face) type of contact. Mainly convivial social interactions with friends or acquaintances that they had before the COVID-19 pandemic had been lost because it was no longer possible to meet in person. Especially the type of contacts for sociability had been lost and were especially missed. Although most participants planned to regain contact with these network members again, it was also mentioned that this was difficult because they had not been in touch for a while. Some participants had recently lost their partner and mentioned that their social network members did not contact them (so much) after their partner had passed away.

#### Preferences mode of contact

Most participants preferred in-person contact over contact by telephone or by internet, as with in-person contact you can see and touch each other, and you can see the facial expression and posture of the other person:* “That personal contact, really seeing each other is preferable, absolutely. And of course, video calling is even more personal than regular calling, in that you can look each other in the eye. But when you are in the room together that gives a different feeling. It is the body language probably, and it is a more complete picture when you sit together in the room. That feels different.” (P5, male, 60–64y, retired/not working, not living alone)*. However, some mentioned being pleasantly surprised with the online contact they have had during the COVID-19 pandemic.

In groups, in-person contact was preferred because online contact was very chaotic for participants. However, some participants also preferred telephone contact when confronting difficult topics or because telephone contact is quicker sometimes. Online messaging was only preferred for quick, short messages.

#### Groups or individual contacts

Some preferred interactions in a group setting (relatively small groups), whereas others preferred bilateral contact. The former was preferred for conviviality, and the latter for discussing more serious matters: *“It depends on the topic: more serious matters are better discussed one on one, while doing fun activities or just for conviviality group setting is also fine.” (P13, male, 65–69y, retired/not working, not living alone).*

Many of the participants reported the importance of contacting persons who have the same interests or life experiences. Most reported that age and sex did not affect the quality of social contact. However, participants did report not preferring network members that were a lot younger or older than themselves. One female participant discussed personal matters preferably with women rather than men. Some participants would like to practice a sport together or go for a walk together with a group. For most participants, meeting new persons to perform these activities was not perceived as a barrier.

Most participants would prefer to have more contacts for conviviality and serious conversations. Some reported only the need for more conviviality. Most were open to adding new contacts to their network, while others were not as they were satisfied with their current social network. A desire for more spontaneous contacts (as was the case before the COVID-19 pandemic) was expressed. Lastly, participants mentioned enjoying providing support to others and they also stated to feel better themselves when providing support. An overview of all coded themes can be found in Appendix B.

## Discussion

Our current study assessed the social network structure and function of older adults in the Netherlands during the COVID-19 pandemic, how their social network influences their health behaviors, and how older adults would prefer to strengthen their social network. Social networks of older Dutch people consist of various relationship types such as family, friends, (former) colleagues, neighbors, and acquaintances, who provide social support. Type of relationships also determine the type of social support. Received social support is an important aspect of health behavior; participants were motivated to exercise more often or eat healthier. Preferred strengthening of social networks included in-person bilateral contact or in-person contact in small groups.

For the here interviewed older adults, family and friends played an important role in the provision of informational and practical support. Previous studies have shown that older adults often have a social network focused on close family and friends (Fiori et al. 2007). Especially the partner/spouse, but also family members are an important source of social support (Stuifbergen et al. 2008; van Groenou and van Tilburg 1996). In our current study, family members were mentioned more frequently to provide support when being ill than a partner/spouse. A possible explanation could be that in the case of COVID-19, the partner could not leave the house either. Also, since both the respondent and partner are older adults, family members could have done their grocery shopping to minimize exposure to the virus. For practical support regarding jobs around the house, family members were mentioned more often instead of partner/spouse. The partner (if present) was often older, jobs could be more difficult to perform due to older age and done by family members.

Emotional support was most often received from a partner or spouse and family members. Especially a partner or spouse is an important emotional supporter among older adults and might even reduce loneliness (Chen and Feeley 2014), whereas emotional support received from family members might have a positive association with well-being (Merz and Huxhold 2010). Most participants confided in their partner or spouse, family members or close friends when they needed to discuss important topics, rather than their other friends or acquaintances. The latter were mainly for conviviality, indicating the importance of diverse relationships within a social network for diverse types of social support.

During the COVID-19 pandemic, participants received all types of social support from network members. In-person contact for social support was strongly preferred and participants occasionally disregarded COVID-19 preventive measures, such as social distancing, to receive in-person social support. However, social support was mostly provided via online contact (telephone or an online platform) during the COVID-19 pandemic, indicating online contact could be a temporary alternative (Marinucci et al. 2022).

Most participants were positively influenced by their network members regarding their health behavior. By exercising together, participants were motivated to exercise more often than they would do alone. The most common reason was the joy they experienced when exercising together, which could be considered as a type of social positive reinforcement. Mostly, participants exercised with a partner, confirming the importance of partners in exercising behavior, in line with a mixed method study among older adults (Schlenk et al. 2021). Exercising together with others might also function as a coping mechanism to deal with stressful life events such as the COVID-19 pandemic and the lockdown periods (Hailey et al. 2022).

Participants were self-deciding considering their diet because they had the skills or knowledge to do so. These are personal factors that influence dietary behavior. However, partners sometimes positively influenced diet patterns by making healthy food choices or cooking for participants. Positive influence operates by providing participants access to resources such as healthy food or information about diet. Partners improve the quality of the diet of older adults, as living together with a partner creates an incentive to cook (healthy) (Dean et al. 2009). However, living alone was not associated with quality of diet (Jackson et al. 2022).

Network members, usually family members or friends, can also negatively influence dietary patterns. The unhealthy behavior of network members complicates maintaining healthy behavior for participants. Negative influence mainly operates through modeling and providing access to (unhealthy) resources. However, several studies have assessed that family members and friends positively influence the diet quality of older adults (Bloom et al. 2017; Conklin et al. 2014), whereas another study concluded that especially family members and friends make a person eat more when eating together (Ruddock et al. 2019; De Castro 1994). Nevertheless, the importance of family members and friends could differ for older adults in comparison with younger adults.

Overall, participants were satisfied with their social network structure and function. However, due to COVID-19 preventive measures, including social distancing, more in-person contact with network members was desired. The lack of social contact made some participants (who live alone, but also those who do not live alone) feel lonely, which is in line with other studies assessing loneliness during the COVID-19 pandemic (Green et al. 2022). Especially conviviality with friends and acquaintances was lacking, indicating that conviviality mainly operates through in-person contact. A Dutch study assessed that older adults missed in-person social contact with friends and acquaintances, which could not be replaced by telephone or digital conviviality (Baâdoudi et al. 2021). Others enjoyed the time that they had to spend alone during the lockdowns because they finally had the time to do their own hobbies (for example reading).

Regarding the strengthening of social networks, participants mentioned both support (emotional support (discomfort), and practical support) and conviviality to be important aspects of a social network. Participants preferred group contact for conviviality and one-on-one contact for discussing important matters. This indicates that especially conviviality was missed the past year, since group contact was not possible during the past year due to COVID-19. A systematic review of interventions targeting loneliness and social isolation in older people assessed that both individual and group interventions could be effective (Poscia et al. 2018).

### Recommendations

As social networks are of key importance in healthy behaviors (such as healthy dietary patterns or exercising), networks should be part of health promotion strategies. In our current study, received social support through different types of relationships was recognized as an important aspect of a social network. Social networks can be enhanced by strengthening the bond between existing network members and mobilizing the network for social support. Online contact could be proposed as a first connection to meet new persons based on shared interests or the same life experiences to gain new network members and thereby enlarge the social network. Bilateral in-person contact would be best for discussing important matters, whereas (smaller) group contact would be most suitable for conviviality. 

### Strengths and limitations

This study is unique because it is the first study that uses qualitative research to question structural and functional aspects of a social network and to identify the role of the network in lifestyle and health. Furthermore, multiple researchers were independently included to code and interpret the data, thereby increasing the objectivity of the data. Some limitations should be acknowledged. Participants were interviewed about their social network members in the past year. Due to a larger period, participants might be susceptible to recall bias (Wang and Cheng 2020), which could result in an underestimation of structural and functional network aspects. Lastly, nonresponse bias might have occurred, since the majority of the participants were satisfied with their social network (Prince 2012). Those who are less satisfied with their social network might not have participated in our current study because they do not feel the need to talk about these matters.

## Conclusions

Family members, partners, and friends played an important role in the provision of social support during the COVID-19 pandemic. The type of relationship is also important for the type of social support, emphasizing the need for a diverse social network. Older adults’ partners influence health behaviors such as exercising and dietary patterns. Social support and conviviality are considered important aspects of social networks for older adults. Future steps to promote healthy behavior in older adults should include social support roles of existing network members or connecting with new network members for conviviality.

## Data Availability

The data of this study contain potentially identifying and sensitive participant information. Due to the General Data Protection Regulation, it is not allowed to distribute or share any personal data that can be traced back (direct or indirect) to an individual. In addition, publicly sharing the data would not be in accordance with participants’ consent obtained for this study. Therefore, data used and/or analyzed during the study are available from the head of the data archiving of the Public Health Service South Limburg on reasonable request. Interested researchers should contact the head of the data-archiving of the Public Health Service South Limburg (Helen Sijstermans: helen.sijstermans@ggdzl.nl) when they would like to re-use data.

## Appendices

### Appendix A. Topic list

**Participant demographics**

•Age

•Living situation (living alone or living together)

•Marital status

•Employment status

•Health status

**Social network structure**

•Type of relationship (partner, family, neighbors, housemates, colleagues, acquaintances, care provider (GP or caregiver))

•Proximity

•Mode of contact (in-person, by telephone, or by internet/chatting)

•Frequency of contact

•Preferred mode of contact

**Social network function**

•Informational support (advice)

•Emotional support (discomfort)

•Emotional support (important decisions)

•Practical support (jobs)

•Practical support (illness)

•Happy/difficult relationship

•Taking care of another person

•Too little support

**Impact of social networks**

Health behaviors

•Exercise

•Diet

•Social participation

Loneliness

•Felt lonely or not

•Reason for feeling lonely

•Needs to not feel lonely in future

**Intervention**

•Important aspects of social network

•Advantages and disadvantages

•Consideration of advantages and disadvantages (choice in consideration)

•Change existing relationships

•Add relationships

•Remove relationships

•Preferences for intervention
